# Conflicting Priorities Between Risk Management and Treatment of Schizophrenia in Swiss Forensic Services—A Case Report

**DOI:** 10.3389/fpsyt.2018.00680

**Published:** 2018-12-06

**Authors:** Sarah Steinau, Nathalie Brackmann, Ulf Sternemann, Nikola Biller-Andorno, Elmar Habermeyer

**Affiliations:** ^1^Department of Forensic Psychiatry, University Hospital of Psychiatry, Zurich, Switzerland; ^2^Institute of Biomedical Ethics and Medical History, University of Zurich, Zurich, Switzerland

**Keywords:** forensic psychiatry, deprivation of liberty, ethics, therapeutic measures, incarceration, correctional psychiatry

## Abstract

The Swiss Criminal Code provides measures for mentally-ill offenders focusing on their need for treatment. This may lead to the deprivation of the patient's liberty up to several years. Under certain circumstances the mentally-ill offender can be sentenced to an indefinite incarceration. This case presentation we will describe a forensic psychiatric patient diagnosed with schizophrenia who was ordered an indefinite incarceration in Switzerland after he had been sentenced to 8 years of imprisonment for a deliberate killing. Initial presentation of symptomatology included formal thought disorders and negative symptoms such as affective flattening and alogia. Due to a scarcity of adequate treatment sites in the 90s and lack of scope for risk assessment and management, the patient could only be treated within highly regiment prison environments in the past. There, the patient's treatment concept primarily focused on short-term psychiatric care instead of providing an adequate treatment plan that would have been essential for the patient's improvement of chronic symptoms. This case description aims to present some of the fundamental issues observed in the forensic mental health system, where strong efforts are made to balance risk management and the treatment of severe mental health disorders. We will put the patient's own course of treatment and his progress within the penal system into context with ethical challenges in the forensic and correctional services and will provide potential recommendations for future research in the field of forensic psychiatry.

## Introduction

The treatment of delinquent patients with schizophrenia is a challenging endeavor at the interface of the health and justice system (1). There is an increased risk for violent behavior in schizophrenic patients (2–4) requiring a secure treatment setting that neither psychiatric institutions nor prison environments could ensure in the past. This has led to significant changes within national treatment services in Switzerland, improving in-patient care for forensic psychiatric patients by allowing disorder-specific therapy of offenders in a high-secure setting (e.g., center for Forensic Psychiatry Rheinau).

In conformity with the Swiss Criminal Code (CC), an offender can be sentenced to a therapeutic measure by the Swiss court. This presupposes that the offender suffers from a mental health disorder associated with the committed felony and that further risks of such offenses can be prevented or reduced by the treatment itself [article 59 CC; (5)]. Release on parole, lasting between 2 and 5 years, can be ordered as soon as a decreased risk for violent or delinquent behavior at liberty is expected. After the expiry of the probationary period the offender is granted final release. If the treatment in accordance with article 59 [CC; (5)] does not promise significant treatment results and if the dangerousness of the mentally disordered offender is evaluated as too high a risk for others, an indefinite incarceration can be executed [article 64 CC; (5)], given that the offender carries a maximum sentence of 5 or more years. If during the execution of the indefinite incarceration the offender fulfills the requirements for an in-patient therapeutic measure, the sanction can be retrospectively modified and converted into a therapeutic measure [article 65 CC; (5)].

Here, we describe the case of a 56-year old forensic psychiatric patient who was initially sentenced to 8 years of imprisonment. On the basis of a severe schizophrenia and difficulties in the management of his security risk, he was sentenced to an indefinite incarceration in the 90s. After a duration of approximately 15 years, the patient's sanction was modified into an in-patient therapeutic measure according to article 59 [CC; (5)]. In this case presentation his course of treatment is analyzed and discussed. In addition, the patient's experiences within the penal system are put into context with ethical challenges within the forensic mental health system and prison environment.

## Case Description

With the verdict of a Swiss court in the late 1980s, the patient was sentenced to 8 years of imprisonment for a deliberate killing at the age of 26. Shortly after, his custodial sentence was partially suspended in order to take sufficient account of his culpability [article 43 CC; (5)]. According to a first forensic expert evaluation in 1989, the patient had been evaluated as impaired in terms of legal culpability due to a schizophrenic episode. Recommendations given included immediate in-patient treatment prior to the court trial.

After the patient had tried to escape multiple times and initial treatment attempts did not show any significant effects, the patient was ordered an indefinite imprisonment according to article 64 [CC; (5)], primarily for safeguarding purposes. This was accompanied by basic psychopharmacological and delinquency-oriented psychotherapeutic care.

In the late 2000s, the patient's sanction was modified [article 65 CC; (5)] and retrospectively converted into an in-patient therapeutic measure according to article 59 [CC; (5)] by the former court. Up to the conversion of the indefinite detention into a therapeutic measure, the patient had been receiving indefinite incarceration in various prison environments for more than 15 years. He was ordered another 8 years of sanction under article 59 [CC; (5)] and eventually received conditional release status for a probationary period of 5 years, until 2020.

### Diagnoses and Course of Treatment

Our patient had grown up under socio-economically beneficial conditions. He was described as a quiet and self-effacing child with an above-average intelligence. At the young age of nine, he intentionally raised fire; further smaller offenses included simple thefts. Around his early twenties, he prematurely terminated his apprenticeship in Switzerland, showing initial psychopathological symptoms of a schizophrenic prodrome. A few years later he was admitted to a psychiatric unit with depressive symptoms, anxiety and comorbid substance abuse, just days prior to the index offense. According to the forensic expert evaluation at the time of the trial, the patient presented formal thought disorders, such as poverty of speech, illogicality and neologism. Furthermore, he showed early signs of negative symptoms including asociality, alogia, and affective flattening. The patient was diagnosed with a paranoid schizophrenia (ICD-10 F20.0) according to the International Classification of Diseases [(6); corresponding to schizophrenia, paranoid type, DSM-IV-TR 295.30 (7)] and comorbid substance use disorder, particularly alcohol abuse (ICD-10 F10.1; DSM-IV-TR 305.00) and cannabis abuse (ICD-10 F12.1; DSM-IV-TR 305.20) prior to the offense. There were no signs of dependence and substances were successfully withdrawn when the patient was first sentenced to prison without any known drug relapse.

In the initial course of the mental illness, the patient presented himself malcompliant. He irregularly refused psychopharmacological treatment and tried to escape multiple times. It was not until some years later, that he adapted his behavior while at the same time showing a progressing chronification of the illness around 1995. The complex psychopathological symptomatology was then dominated by the patient's negative symptoms and a drug-induced parkinsonism. This consequently led to the patient's severely impaired mimic and gestural expressiveness, reduced and quiet speech, limited eye contact and a reduced psychosocial level of functioning. Hence, psychotherapeutic approaches and psychosocial treatment and support did only show limited success, leading to aggravated treatment conditions. After the implementation of the therapeutic measure according to article 59 [CC; (5)], the therapeutic approach solely included the patient's physical and psychiatric support as well as relief in the management of daily challenges. After having spent almost 30 years in prisons and psychiatric institutions for interventional purposes, the patient did not have any relevant social contacts or relationships. In 2018, he passed away at the age of 56 from the consequences of a severe physical condition.

### Evaluation of Therapeutic Efficacy

Despite the administration of therapeutics, the course of treatment only showed partial success. Due to the side effects caused by the long-term intake of conventional antipsychotic agents, the patient was significantly hindered in coping with everyday challenges. Initial attempts to escape and the associated higher risk of violence led to the execution of an indefinite incarceration in 1990. Positive symptoms or malcompliance were no longer observed after 1995 and had been fully replaced with a negative symptomatology. This was interpreted in the context of the severe chronification of his mental illness with a poorer physical and mental state compared to the preceding years.

## Discussion

The present case describes a forensic psychiatric patient who spent almost 30 years under institutional control. The course of the indefinite incarceration and its subsequent conversion into an in-patient therapeutic measure was characterized and dominated by a severe chronic schizophrenia with a negative symptomatology and drug-induced parkinsonism. Due to his mental health disorder, the patient's character and behavior reflected the loss of normal functions such as losing interest, not being able to experience pleasure, and reduced social drive or action. Additionally, the patient was severely restricted in his movements due to extrapyramidal symptoms causing dyskinesia and symptoms similar to a Parkinson's syndrome.

In Switzerland, the court may sentence someone who committed a serious crime, such as murder, to indefinite incarceration [article 64 CC; (5)]. This sanction can be ordered, if the treatment of the offender is hardly accompanied by relevant success or if he is deemed untreatable. Another reason may be the protection of society from criminals with a high risk of relapse for further severe offenses. This poses noteworthy challenges to legal decision makers. Of utmost interest for the outlined case are the considerations pertaining to the dilemma of ensuring a suitable psychiatric and specialized treatment concept on the one hand while guaranteeing the safety of the public on the other hand. According to an early medical report by a psychiatric institution in 1993, the patient's case already then caused a “dilemma” to psychiatrists, because medical professionals recommended an in-patient treatment setting that could at that time not be implemented due to the suspected high risks. This resulted in the fact that our patient spent most of his past life years in correctional institutions, rather than receiving an adequate treatment concept in the management of a severe and chronic mental health disorder, such as schizophrenia. Thus instead of receiving a treatment according to the risk-need-responsivity model (8), the patient was merely incarcerated and only received little interventions that would have further reduced his risk to recidivate. This may have then allowed the patient to be released earlier or to be granted the opportunity for probation, respectively.

Although community and correctional facilities share similar mental health services, correctional settings tend to be more restrictive in terms of bureaucratic obstacles, to have less well instructed or trained employees and to show a slower execution of therapeutic steps in the management of a psychiatric crisis or psychotic episodes. Criminals sentenced under the aforementioned statute only receive scarce psychiatric care that does not focus on the treatment of the mental illness. It therefore remains unclear how continuous the treatment and intake of therapeutic antipsychotic agents was in the 1990s and how strong an effect it would have had on the patient's treatment course and outcome had it been administered in a professional clinical setting. Hence, a remitted psychopathological mental state may have led to a sooner release status due to adequate risk management. This could have prevented the patient from spending up to almost 30 years in the executional system of penal sentences and justice, compared to his initial sanction of 8 years of imprisonment.

These considerations lead to one of the fundamental problems that can be observed in forensic psychiatry, namely a scarcity of adequate treatment sites. The former lack of scope for managing the treatment of mentally ill and potentially violent offenders could only be combatted by falling back on prison environments. This “shifting” of delinquent patients with high treatment demands into regimented settings may have failed to prioritize effective mental health services. Prison environments bear defining difficulties for patients who may lack social competences and show deficient abilities to cope with the stresses of being imprisoned. Additionally, prison rules and regulations usually apply to all inmates equally, with treatment being subordinated to security procedures. Hence, the patient's treatment concept may only focus on the management of short-term psychiatric care in emergency situations, losing sight of long-term treatment outcomes within prison environments. In the patient's case his physical and psychopathological state in terms of a proceeded negative symptomatology should have generally led to an earlier relocation from the prison environment to an adequate psychiatric institution. If the patient had shown e.g., delusional symptoms or signs of verbal or physical aggression, one might have been more aware of his needs. Instead, the patient—due to his psychopathological symptoms such as psychomotor retardation and affective flattening—had shown a rather imperceptible behavior in contrast to other inmates that might have just not been perceived as a disturbing or “pathological” behavior. Hence, one of the reasons for the patient's long-lasting incarceration could be that he had simply been forgotten within the prison environments. Yet, personal data on experiences of mentally ill offenders in prisons are scarce and disadvantageous conditions only assumed, lacking the scientific information about what may generally and specifically matter to imprisoned patients dealing with mental health disorders.

Another ethical issue concerns whether or not our patient could really have been released earlier, reducing his deprived years of liberty. According to medical and legal documentation, the patient did not show any significant aggressive or impulsive behavior, especially after 1995. Then again, his mental health status was discussed to be an important key factor for not granting release. Additionally, the patient suffered from comorbid multiple substance abuse, affecting the threshold of aggression (9). Clearly, the patient's risk for relapse was high and so was the higher risk for associated violent behavior (10). Taking the manifestation of the patient's mental health disorder and the severity of his crime into account, this clearly shows how strongly the forensic mental health system suffers from balancing treatment on the one and security on the other hand. Risk factors, such as a criminal record, severe mental health disorder and substance abuse, are essential for the determination of re-offense rates (11). Yet, the individual likelihood of re-offending should not be lost out of focus. In the case presented the assumption of a comparable risk compared to that observed re-offense rate in a research sample may have not met the patient's needs. In that case, the missing of an established and appropriate psychiatric institution might have contributed to the aggravation of negative symptoms and manifestation of the chronic illness, respectively.

Furthermore, risk evaluations are mainly required by others in order to serve their wishes for protection from the patient. The patient himself has little or no say at all in the outcome of the assessments that take place. When directly asked about it in a psychiatric evaluation in 2011, the patient stated, over a total of 25 years, that it was the justice system not having left any other possibilities open. In addition, the medical staff may morally justify the decision made, e.g., to restrict the autonomy of the patient because past events have clearly demonstrated the patient's competency to place his own needs ahead of those of others—as could be seen by the patient's index offense in the late 1980s. Hence, psychiatrists could be seen to be acting more in the service of their institutions.

Our case may stress the importance to comprehensively understand individual's needs for diagnostic and therapeutic options as well as the assessment of violence risk in the context of incarceration. Although our patient had not been in a state of torture, he had clearly been deprived of his liberty as well as from a continuous and appropriate psychiatric treatment, as formerly stated and suggested by psychiatrists in the early 1990s. This might only be justified by the lack of appropriate psychiatric institutions at that time, ensuring adequate and specialized in-patient treatment for schizophrenic patients and trained employees while at the same guaranteeing high security standards.

This former “shifting” of treatment places has experienced an improved infrastructural organization within the recent years. Yet, adequate treatment sites for offenders with mental health disorders are still scarce. Sustainable results in terms of a just distribution of treatment options and quality demand specialized care and the availability of appropriate treatment sites or psychiatric institutions with high security standards, respectively. In accordance with that, the offender's treatability should be assessed more regularly to avoid malpractice and improve the patient's mental health and physical status, potentially minimizing the time spent in a deprived setting. This may be implemented by allowing therapeutic measures to be less restrictive in terms of creating more broadly based regulatory options within the system of penal sentences and justice. Whereas, treatment in general psychiatry strives to ensure individualized therapy that goes beyond common guidelines, its subspecialty may even suffer to guarantee sufficient treatment standards for mentally ill offenders, most of whom are being treated for severe and chronic mental health disorders. Therefore, it seems essential to expand treatment availability in terms of capacity, positively contributing to a better treatment concept that meets today's medical and individual challenges in the therapy of e.g., chronic schizophrenia in a cohort of delinquent patients. Furthermore, it may be worth assessing patient experiences in both prison environments and adequate psychiatric institutions, as done scientifically for various health conditions with qualitative studies of people's experiences (12) in the UK (13) or Germany (14). Listening to patients' voices is accompanied by a growing recognition of personal experiences as a relevant source of information for both ethics in the health care system and policy debates. Obtaining narrative accounts and getting an insight into personal front row experiences, especially narratives of those patients living under regimented conditions, may enable a better responding to the needs of patients within the forensic mental health services and prospective outcomes. This, in turn, may serve to improve risk management and to reduce the number of cases in which mentally ill prisoners are merely incarcerated without adequate treatment options.

### Limitations

The present case description tries to highlight potential issues that arise from the interface of forensic services and the penal system with special emphasis on ethical concerns. Yet, a challenge in evaluating old case files poses the varying degrees of documentation due to outdated quality assurance guidelines. The information given in the present case files, especially on therapeutic strategies, was sometimes vague and limited. Only occasionally, therapeutic concepts, such as the execution of a delinquency-oriented therapeutic approach, were mentioned, but more specific information about duration, content or adherence was missing. It therefore remains unclear to what extent psychotherapeutic concepts were actually implemented into the treatment plan, though it can be assumed that, at least, the initial treatment did not successfully target the patient‘s needs.

## Ethics Statement

Written informed consent was obtained prior to the publication of this case report by a senior physician, who clinically assessed the patient's capacity to consent to participation. The patient then gave written consent on the basis that disclosed material would be anonymised and unidentifiable. Identifying details have therefore been removed from the report where necessary.

## Author Contributions

SS and NB contributed equally to the design and concept of the work, data analysis, interpretation, and drafted the manuscript. SS collected the data. US and NB-A contributed expertise and critical revision of the final manuscript. EH contributed to the concept of the work, the drafting of the article and revision of the final manuscript.

### Conflict of Interest Statement

The authors declare that the research was conducted in the absence of any commercial or financial relationships that could be construed as a potential conflict of interest.
